# Potential therapeutic benefits of unconventional oils: assessment of the potential *in vitro* biological properties of some Rubiaceae, Cucurbitaceae, and Brassicaceae seed oils

**DOI:** 10.3389/fnut.2023.1171766

**Published:** 2023-04-21

**Authors:** Florinda Fratianni, Giuseppe Amato, Vincenzo De Feo, Antonio d'Acierno, Raffaele Coppola, Filomena Nazzaro

**Affiliations:** ^1^Institute of Food Sciences, National Research Council of Italy, Avellino, Italy; ^2^Department of Pharmacy, University of Salerno, Fisciano, Italy; ^3^Department of Agricultural, Environmental and Food Sciences, University of Molise, Campobasso, Italy

**Keywords:** antioxidant, vegetable oils, biofilm, fatty acids, cholinesterases inhibition, tyrosinase inhibition, anti-inflammatory (activity)

## Abstract

**Introduction:**

Seed oils are versatile in the food sector and for pharmaceutical purposes. In recent years, their biological properties aroused the interest of the scientific world.

**Materials and methods:**

We studied the composition of fatty acids (FAs) and some *in vitro* potential therapeutic benefits of five cold-pressed commercial oils obtained from broccoli, coffee, green coffee, pumpkin, and watermelon seeds. In particular, we assayed the antioxidant activity (using diphenyl-1-picrylhydrazyl (DPPH) and azino-bis (3-ethylbenzothiazoline-6-sulfonic acid (ABTS) assays). In addition, through the fatty acid composition, we calculated the atherogenicity index (AI) and thrombogenicity index (TI) to evaluate the potential impact of such oils on cardiovascular diseases. Furthermore, we assessed the *in vitro* anti-inflammatory capacity of the oils (evaluated through their effectiveness in preventing protein degradation, using bovine serum albumin as protein standard) and the ability of the oils to inhibit *in vitro* activity of three among the essential enzymes, cholinesterases and tyrosinase, involved in the Alzheimer's and Parkinson's neurodegenerative diseases. Finally, we evaluated the capacity of the oils to inhibit the biofilm of some pathogenic bacteria.

**Results:**

The unsaturated fatty acids greatly predominated in broccoli seed oil (84.3%), with erucic acid as the main constituent (33.1%). Other unsaturated fatty acids were linolenic (20.6%) and linoleic (16.1%) acids. The saturated fatty acids fraction comprised the palmitic (6.8%) and stearic acids (0.2%). Broccoli seed oil showed the best AI (0.080) and TI (0.16) indexes. The oils expressed a good antioxidant ability. Except for the watermelon seed oil, the oils exhibited a generally good *in vitro* anti-inflammatory activity, with IC_50_ values not exceeding 8.73 micrograms. Broccoli seed oil and green coffee seed oil showed the best acetylcholinesterase inhibitory activity; coffee seed oil and broccoli seed oil were the most effective in inhibiting butyrylcholinesterase (IC_50_ = 15.7 μg and 20.7 μg, respectively). Pumpkin and green coffee seed oil showed the best inhibitory activity against tyrosinase (IC_50_ = 2 μg and 2.77 μg, respectively). In several cases, the seed oils inhibited the biofilm formation and the mature biofilm of some gram-positive and gram-negative bacteria, with *Staphylococcus aureus* resulting in the most sensitive strain. Such activity seemed related only in some cases to the capacity of the oils to act on the sessile bacterial cells' metabolism, as indicated by the 3-(4,5-dimethylthiazol-2-yl)-2,5-diphenyltetrazolium bromide (MTT) colorimetric method.

## Introduction

Pumpkin (*Cucurbita maxima* Duchesne) and watermelon (*Citrullus lanatus* Matsum. & Nakai), belonging to the Cucurbitaceae family, are among the most widely cultivated plants, growing in temperate, tropical, and subtropical areas. Rich in vitamins and other important phytochemicals (such as carotenoids, polyphenols, and anthocyanins), they can be consumed as fresh products or in processed forms (for instance, watermelon can be eaten as juice, puree, and jam; pumpkin can be consumed as cooked, roasted, jam, and cream). In both cases, some portions of the fruits, particularly peels and seeds, are discarded; these by-products are economically unprofitable due to their costs for drying and transport (1–5). Green coffee and coffee (*Coffea arabica* L.—Rubiaceae) are consumed as beverages in all parts of the world. Coffee beverages are also considered an important source of therapeutic phytochemicals, such as caffeine, with anti-cancer, antioxidant, and anti-inflammatory activity; they can also fight neurodegenerative, cardiovascular, and dysmetabolic diseases (6). Broccoli (*Brassica oleracea* L.), belonging to the Brassicaceae family, is one of the most commonly cooked-consumed vegetables in several countries in the world and is known not only for its sensory characteristics but also for its nutritional value due to the presence of secondary metabolites, such as glucosinolates, isothiocyanates, and indoles (7). In recent years, fruit seeds have received attention due to their valuable nutritional and medicinal properties. They can give oils with many bioactive metabolites and natural antioxidants (8). Cucurbitaceae seed oils can help treat several diseases due to their multiple effects, and in many cases, they are suitable for human needs (4, 9). Watermelon seeds represent an excellent dietary oil source with biological properties (2, 10). The by-products of coffee, including coffee oil, have beneficial effects (11). Cucurbitaceae and Brassicaceae seed flours can enhance the total number and composition of gut bacteria and demonstrate excellent antioxidant and anti-inflammatory activities, suggesting their use in improving human health (12). The coffee seed oil contains terpenes (13), fatty acids, particularly linoleic and palmitic, and sterols (sitosterol, campesterol, and cycloartenol). Roasted coffee beans does not modify triglycerides; on the contrary, the process causes an increase in fatty acid concentration (14). Coffee oil exhibited different beneficial effects, such as anti-cancerous, anti-inflammatory, anti-bacterial, anti-viral, anti-diabetic, and anti-atherosclerotic (15) and also exerts protective effects against nephrotoxicity in animal models (16). Broccoli seed oil demonstrated inhibitory activity against the growth of different gram-positive and Ggam-negative bacteria (17), and, like other Brassicaceae, it can exhibit multiple health functions (18) indeed. Broccoli seed oil was recently used to develop innovative green materials due to its high content of long-chain fatty acids, such as erucic acid. However, due to the high content of omega-9 fatty acids, broccoli seed oil could not be used as food (19). Recent studies demonstrated that in mixture with oleic acid (giving rise, for instance, from EVO oil) is often used as a dietary treatment for managing some neurodegenerative diseases without toxic effects (20, 21). Given these facts, the purpose of this article was to study the fatty acid composition and some *in vitro* biological properties of broccoli, coffee, green coffee, pumpkin, and watermelon seed oils, to evaluate *in vitro* their potential antioxidant and anti-inflammatory properties and their capacity to inhibit the cholinesterases and tyrosinase enzymes involved in some neurodegenerative diseases, as well as to impede or limit the biofilm formed by some gram-positive and gram-negative pathogenic bacteria, of interest both for food and health sectors.

## Materials and methods

### Oils

A total of five commercial seed oils of broccoli, green coffee, coffee, pumpkin, and watermelon were bought from a local market. As specified by the manufacturers, the seeds were cold-pressed, avoiding solvents. Samples were stored at 4°C, avoiding exposure to light until the analysis.

### Determination of fatty acids profile

Fatty acid methyl esters (FAMEs) were obtained by trans-methylation following the method of El Riachy et al. (22). Chromatographic separation was performed using an HP-5MS capillary column (30 mm, 0.25 mm, and 0.25 μm) and helium as carrier gas (1 mL/min). The FAMEs injection (1 μl, 10% in CH_2_Cl_2_, v/v) was carried out in split mode (50:1). The injector temperature was 250°C, whereas the detector temperatures were 280°C and 180°C for flame ionization detector (FID) and mass spectrometry (MS), respectively. For the gas chromatography–mass spectrometry (GC-MS) analysis, the ionization voltage, the electron multiplier, and the ion source temperature were set at 70 eV, 900 V, and 230°C, respectively. We used the following elution program: 220°C for 6 min, increased to 270°C at 3°C/min, and held at 270°C for 4 min. The compounds were identified by calculating their Kovats retention index with respect to the reference standard. The analyses were performed in triplicate, and the values reported are the mean ± SD of three experiments.

The atherogenicity index (AI) and thrombogenicity index (TI) were calculated according to Khalili Tilami and Kouřimská (23), using the following equations:


$$\begin{array}{l} IA=\left[C12:0+\left(4^{*}C14:0\right)+C16:0\right]\;/\Sigma UFA \end{array}$$

$$\begin{array}{l} IT=(C14:0+C16:0+C18:0)/[(0.5\times \Sigma MUFA)\\ \;+(0.5\times \Sigma n-6PUFA)+(3\times \Sigma n-3PUFA)\\ \;+(n-3/\;n-6)] \end{array}$$

### Evaluation of the antioxidant activity

For the evaluation of the antioxidant activity, we followed the protocol of Fratianni et al. (24). The oils were mixed with acetone (Sigma, Milano, Italy) (1:1 v: v); 1% methanol-HCl was added (1:2, v: v) to each mixture after 1 h of storage at room temperature. Samples were stored for another hour at room temperature and subjected to centrifugation at 13,000 rpm for 5 min. The supernatant was recovered, and the residue was subjected again to the extractive process. The two supernatants were pooled and used for the analysis.

### Diphenyl-1-Picrylhydrazyl (DPPH) test

The free-radical scavenging activity of the seed oils was determined in microplates through the diphenyl-1-Picrylhydrazyl (DPPH) test (25). Samples were previously diluted to 1:1 vol/vol in dimethyl sulfoxide (DMSO), and then mixed with 303 μl of a methanol solution of DPPH (153 mM). The absorbance was measured at λ = 517 nm (UV-VIS spectrophotometer Cary Varian, Milano, Italy). The absorbance of the DPPH radical, without the sample, was used as a basis. The amount of samples (micrograms) needed to inhibit the activity of 1 ml of DPPH at 50% indicated the IC_50_ (μg). The tests were performed in triplicate, and the results were expressed as the mean ± SD.

### 2,2′Azino-bis (3-ethylbenzothiazoline-6-sulfonic acid) (ABTS) test

The antioxidant activity was assessed through the 2,2′Azino-bis (3-ethylbenzothiazoline-6-sulfonic acid) (ABTS) test (26). The ABTS diammonium salt, potassium persulfate (dipotassium peroxidisulfate), 6-hydroxy 2,5,7,8-tetramethylchroman-2-carboxylic acid (Trolox^®^), and LC-grade methanol were purchased by the Sigma–Aldrich Milano, Italy. A measure of 2.5 mM of Trolox^®^ (methanolic daily preparation) constituted the antioxidant stock standard. First, the ABTS and potassium persulfate were suspended in distilled water (final concentration of 7 and 2.45 mM, respectively), mixed, and kept in the dark at room temperature before its use to produce the ABTS radical (ABTS^+^). Next, the ABTS radical solution was diluted with distilled water to have a final absorbance at λ 734 nm=1.00). Finally, samples (final concentrations 0.0001–0.0100 mg/ml) or Trolox^®^ standard (final concentration 0–20 mM) were added to the diluted ABTS+ solution; the absorbance was read 6 min after mixing. The average triplicate determinations were expressed as the samples μmol Trolox^®^ equivalent antioxidant capacity (TEAC) ± SD.

### Anti-inflammatory activity

The method of Fratianni et al. (24) allowed us to evaluate the *in vitro* anti-inflammatory activity by determining the inhibition of serum bovine albumin denaturation (BSA). The reaction mixture (5 ml) included 0.2 ml of a stock solution of 0.5% (w/v) BSA (96% purity, Sigma, Milano, Italy) in 0.05 M Tris–phosphate-buffered saline solution, pH 6.5, 2.8 ml of Tris–phosphate-buffered saline solution, and 2 ml of varying amounts of samples. A measure of 1 ml of BSA containing methanol represented the control. After heating at 72 °C and the subsequent cooling of the mixture, the absorbance was calculated at λ=660 nm. The extract concentration for the 50% inhibition (IC_50_) of the BSA denaturation was compared with the control using diclofenac sodium as the positive control. The tests were made in triplicate, and the results were expressed as the mean ± SD.

### Cholinesterase inhibitory activities

The spectrophotometric method of Ellman et al. (27) allowed us to evaluate the *in vitro* inhibitory activity of acetylcholinesterase (AChE) and butyrylcholinesterase (BChE). All reactions were performed in triplicate in 96-well microtiter plates. Acetylcholine (ACh) was the substrate to assay the inhibition of AChE. The reaction mixture containing 550 μl of sodium phosphate buffer 0.1 M (pH 8.0), 50 μl of the samples, and 5 to 20 ng of AChE (from *Electrophorus electricus* 1,000 units/mg) was incubated for 15 min at room temperature. Samples and the positive control (galantamine, Sigma Aldrich, Milano, Italy) were resuspended in 10% DMSO. The formation of the yellow 5-thio-2-nitrobenzoate anion at λ = 412 nm for 10 min, resulting from the reaction of 5,5-dithio-bis-(2-nitrobenzoic acid (DTNB, 10 μl) with acetylcholine (10 μl), was monitored to evaluate the enzymatic hydrolysis of ACh. The results were expressed as the mean ± SD. Butyrylthiocholine (BCh) was the substrate used to assess the inhibitory activities of BChE. The reaction mixture contained 550 μl of K-phosphate buffer 0.1 M (pH 7.0), 50 μl of sample, and an amount of equine serum BChE (≥10 units /mg protein) ranging from 10 to 50 ng. The components were mixed; after incubation at room temperature for 15 min, we added the substrate BCh and the 5,5-dithio-bis-2-nitrobenzoic acid (DTNB). The hydrolysis of BCh was monitored at λ = 412 nm, following the formation of the yellow 5-thio-2-nitrobenzoate anion after 10 min, giving rise from the reaction of DTNB (10 μl) with butyrylthiocholine (10 μl), released by the enzymatic hydrolysis of BCh. The AChE and BChE *in vitro* inhibitory activities were expressed in terms of the IC_50_ value (μg required to inhibit at 50% the hydrolysis of the substrate). Determinations were performed in triplicate, and the results were expressed as the mean ± SD.

### Tyrosinase inhibition assay

The *in vitro* tyrosinase inhibition test was performed as described by Khatib et al. (28), with minor modifications. First, samples were diluted in DMSO. Next, the reaction mixture (phosphate buffer of 70 μl, pH 6.8), (10 units/ml) and the sample were inserted directly on the 96-microtiter plate and incubated at 37 °C for 5 min; then, 0.5 mM l-dioxyphenylalanine (*l*-DOPA) was added, and the plate was read at λ = 492 or 475 nm after 10 min of incubation at 37°C, using kojic acid as a positive control and phosphate buffer as blank. The inhibitory activity against tyrosinase was evaluated in terms of IC_50_, representing the concentration of samples giving a 50% inhibition of the tyrosinase activity. This estimation was made through the interpolation of the concentration-response curves. All tests were performed in triplicate; the results were expressed as the mean ± SD.

### Antibacterial properties of the oils

#### Microorganisms and culture conditions

The gram-negative bacteria such as *Acinetobacter baumannii* (ATCC 19606), *Pseudomonas aeruginosa* (DSM 50071), and *Escherichia coli* (DSM 8579) and the gram-positive bacteria such as *Listeria monocytogenes* (ATCC 7644) and *Staphylococcus aureus* subsp. *aureus* (ATCC 25923) were the test bacterial strains. The bacteria were cultured in Luria Broth for 18 h at 37°C (35°C for *A. baumannii*) at 80 rpm (Corning LSE, Pisa, Italy) before the analysis.

#### Minimal inhibitory concentration

The minimal inhibitory concentration (MIC) of the oils was evaluated through the resazurin microtiter plate assay (29). Multiwell plates were prepared in triplicate and then incubated at 37°C for 24 h (*Acinetobacter baumannii* was cultured at 35 °C at the same conditions). The MIC value of each oil was evaluated by monitoring the color changes.

#### Biofilm inhibitory activity

We evaluated the oils' capacity to affect the bacterial adhesion following the method of Fratianni et al. (30), in flat-bottomed 96-well microtiter plates. The overnight bacterial cultures were adjusted to 0.5 McFarland (1.5 × 10^7^ cells/ml, Densitometer cell density turbidity 0.3–15.0 McFarland, CAMLAB, Cambridge, United Kingdom), with fresh culture broth before the test. We distributed 10 microliters of the diluted cultures in each well, 10 μg/ml and 20 μg/ml of each sample, and Luria–Bertani broth, to have a final volume of 250 μl/well. Then, the microplates were entirely covered with parafilm tape to avoid the evaporation of the material in the wells and incubated for 48 h at 37°C (*A. baumannii* was incubated at 35°C). After removing planktonic cells, the attached cells were washed two times with sterile phosphate-buffered saline PBS. A measure of 200 microliters of methanol was added to each well and retained for 15 min to fix the sessile cells. After discarding the methanol, each plate was left to dry. A measure of 200 microliters of 2% w/v crystal violet solution added to each well and stained the adhered cells for 20 min. Then, the wells were lightly washed with sterile PBS and left to dry. A measure of 200 microliters of glacial acetic acid 20% w/v let the release of the bound dye. The absorbance was measured at λ = 540 nm. We calculated the percent value of the adhesion with respect to the control (cells grown without the presence of the samples, for whom we assumed an inhibition rate = 0%). Triplicate tests were performed, and the average results were taken for reproducibility.

#### Activity on mature bacterial biofilm

A measure of 10 microliters of the overnight bacterial cultures (adjusted to 0.5 McFarland with fresh Luria–Bertani culture broth) and Luria–Bertani culture broth were added in flat-bottomed 96-well microtiter plates, to have a final volume of 250 μl/well. Then, microplates were fully covered with parafilm tape, to avoid the evaporation of the material in the wells and incubated at 37°C (*A. baumannii* was incubated at 35°C). After 24 h of bacterial growth, the planktonic cells were removed, and two concentrations of the oils, 10 and 20 μg/ml of each sample, and Luria–Bertani broth were added to have a final volume of 250 μl/well. After another 24 h of incubation, the sequential steps of the experiment including the calculation of the percent value of inhibition compared with the untreated bacteria were performed as described above.

#### Inhibition of cell metabolic activity within the biofilm

We also evaluated the effects of the oils, added at the beginning of the bacterial growth and after 24 h of incubation, on the metabolic activity of the biofilm, through the 3-(4,5-dimethylthiazol-2-yl)-2,5-diphenyltetrazolium bromide (MTT) colorimetric method (31, 32). After 48 h of incubation, the culture broth was discarded; then, we added 150 μl of PBS and 30 μl of 0.3% of MTT (Sigma, Milano, Italy), keeping the microplates at 37°C (except for *A. baumannii*, incubated at 35°C). After 2 h, we removed the MTT solution and performed two washing steps with 200 μl of sterile physiological solution. A measure of 200 microliters of dimethyl-sulfoxide (DMSO) added to each well let the dissolution of the formazan crystals, which were measured at λ = 570 nm after 2 h of incubation at 37°C or 35°C (depending on the strain). Tests were performed in triplicate, and the results were expressed as the mean ± SD.

### Statistical analysis

Data were expressed as average ± SD of triplicate measurements. The values of the antibacterial tests were expressed as the mean ± SD and statistically analyzed using a two-way ANOVA followed by Dunnett's multiple comparison test at the significance level of *p* < 0.05, using GraphPad Prism 6.0. (GraphPad Software, Inc., San Diego, California, United States) and MATLAB software (Mathworks, Massachusetts, United States). The analysis also correlated the values of the activities of the oils with the fatty acid composition, using the free software environment for statistical computing and graphics R (https://www.r-project.org/).

## Results and discussion

### Fatty acid composition

The fatty acid profile is presented in Table 1. The unsaturated fatty acids greatly predominated in broccoli seed oil (84.3%), with erucic acid as the main constituent (33.1%). Other unsaturated fatty acids are linolenic (20.6%) and linoleic acids (16.1%). In the saturated fatty acids fraction, we found only palmitic (6.8%) and stearic acids (0.2%). The composition agrees with the available literature, with a major percent presence of unsaturated compounds (32, 33).

**Table 1:** Fatty acid composition (%), index of atherogenicity (IA) and index of thrombogenicity (IT) of the cold-pressed seed oils of broccoli, coffee, green coffee, pumpkin, and watermelon.

	**Broccoli**	**Coffee**	**Green coffee**	**Pumpkin**	**Watermelon**
Myristic acid (C14:0)	-	0.1 ± 0.0	0.2 ± 0.0	-	-
Palmitic acid (C16:0)	6.8 ± 0.7	21.1 ± 0.3	33.3 ± 2.1	10.5 ± 0.6	11.3 ± 1.0
Palmitoleic acid (C16:1)	-	0.2 ± 0.1	0.2 ± 0.1	0.5 ± 0.1	0.3 ± 0.1
Stearic acid (C18:0)	0.2 ± 0.1	47.5 ± 1.1	37.5 ± 1.7	7.9 ± 0.7	20.1 ± 1.2
Oleic acid (C18:1)	15.1 ± 0.8	11.3 ± 0.9	7.5 ± 0.9	31.3 ± 1.1	15.7 ± 1.2
Linoleic acid (C18:2)	16.1 ± 1.0	12.5 ± 1.0	12.5 ± 0.8	42.5 ± 1.3	45.5 ± 2.0
Linolenic acid (C18:3)	20.06 ± 0.9	0.1± 0.0	0.3 ± 0.1	1.3 ± 0.2	2.3 ± 0.3
Erucic acid (C22:1)	33.1 ± 1.2	-	-	-	-
Total	91.3	92.8	91.5	94.0	95.2
Saturated fatty acids	7.0	68.7	71.0	18.4	31.4
Unsaturated fatty acids	84.3	24.1	21.5	75.6	63.8
Ratio saturated/unsaturated	0.083	2.851	3.302	0.243	0.492
Index of atherogenicity (AI)	0.08	0.89	1.6	0.13	0.17
Index of thrombogenicity (TI)	0.16	5.71	6.92	0.48	0.98

The fatty acid composition is expressed as a percentage. - = not detected. The experiments were performed in triplicate and herein is reported the average (± SD).

The fatty acid profiles of coffee and green coffee oils were similar. The main fatty acid was stearic acid with 47.5% (coffee) and 37.5% (green coffee). Palmitic acid was present in lower percentages (21.1% and 33.3%), while myristic acid was present only in traces (0.1% and 0.2%). In both oils, saturated fatty acids were predominated (68.7% and 71.0%, respectively). The roasting did not significantly affect the fatty acid composition, as reported in the literature (14). However, our data differed from those in the literature, particularly for the ratio of saturated/unsaturated fatty acids. Dong et al. reported a lower ratio in several Chinese varieties than that obtained in our study (33, 34).

In pumpkin seed oil, the unsaturated fatty acids represented 75.6% of the total, the most abundant fatty acids being linoleic (42.5%) and oleic acids (31.3%). Our data agreed with the available literature (35).

Watermelon seeds were rich in unsaturated fatty acids (63.8%), with linoleic acid (45.5%) as the main component, followed by stearic (20.1%) and oleic acids (15.7%); among the saturated fatty acids, the most abundant is palmitic acid (11.3%). These results are in agreement with the available literature (36). The broccoli and pumpkin oils resulted rich in unsaturated fatty acids, with possible beneficial effects on human health (37–39).

In recent years, the lipids contained in biological matrices, including vegetable seed oils, have also been studied to evaluate their possible atherogenic and thrombogenic effects (23).

Thus, assessing the indices of atherogenicity (AI) and thrombogenicity (TI) can provide information regarding the different effects that individual FAs could have on human health, particularly the likelihood of an increased incidence of atherosclerosis (40, 41). The incidence of atherogenicity has been associated with various inflammatory and innate immune pathways, and the atherogenic index may be used as a preliminary indication of accelerated atherosclerosis associated with numerous inflammatory pathways (42). AI and TI are one of the most well-known, reliable, and widely used indices for all types of fats and oils that can be used to assess the potential effects of FA on cardiovascular diseases (43). The atherogenic index shows a precursory indication of accelerated atherosclerosis and can support our knowledge of the numerous inflammatory pathways associated with it, while the thrombogenic index shows a tendency for clot formation in the blood vessels and cardiovascular diseases (44). Our results showed variability between the samples both as regards, the atherogenic index and the thrombogenic index. In both cases, broccoli and pumpkin oils revealed promising values with AI of 0.08 and 0.13, respectively, and a TI of 0.16 and 0.48. On the contrary, coffee and green coffee oils exhibited the highest, 0.89 and 1.6 for AI and 5.71 and 6.92 for TI.

In the case of the broccoli seed oil, which contained only traces of stearic acid, the low AI could have been influenced by the simultaneous presence of oleic, linoleic, linolenic, and erucic acids (33.1%), which has a low atherogenic index. On the contrary, the coffee seed oil, showing a high AI, however, contained a very high percentage of stearic acid (47.5%), which notoriously has a low atherogenic index; green coffee seed oil also contained a large percentage of stearic acid (37.5%); however, the lower quantity of stearic acid and oleic acid, associated with the high percentage of palmitic acid, influenced its highest AI. The two Cucurbitaceae seed oils exhibited an AI of 0.13 (pumpkin seed oil) and 0.17 (watermelon seed oil). These values could be attributable to the different percentages of oleic acid, which might influence the healthy function of the two oils more than the stearic acid and palmitic acid. Our data indicated that, except the two *Coffea arabica* oils, the others exhibited IA values similar to or even lower than that shown by the olive oil AI (0.14), (40) avocado seed oil (IA = 0.40), and borage (IA = 0.18) (45). The TI of the broccoli seed oil was also lower than that obtained from red fruit oils (0.23) (46). Therefore, enriching the diet with some of these oils characterized by very low AI and TI, such as broccoli and cucurbit seed oil, could lead the diet to be less atherogenic and induce fewer thrombogenic events, contributing to a decrease in the incidence of risks involving the heart and the circulatory system (40, 45, 46).

### Antioxidant activity

We intended to evaluate the *in vitro* antioxidant properties of the seed oils of broccoli, coffee, green coffee, pumpkin, and watermelon, intentionally of commercial origin, thus accessible to the consumer. The results are shown in Table 2. The antioxidant property of the oils was evaluated through DPPH and ABTS radical scavenging capacity assays, carried out three times to check their reproducibility and to have a wider setting regarding the antioxidant activity. The DPPH test allowed us to quantify the sample-reducing capacity, whether it operated with hydrogen transfer or electron transfer; however, such a technique usually has some limits, such as large sterically bulky molecules incapable of reacting with the reactive part of the radical. On the contrary, the ABTS test offered the opportunity to measure hydrophilic and lipophilic antioxidants over a large pH range.

**Table 2:** Antioxidant activity (evaluated through ABTS and DPPH tests), protective effect against the bovine serum albumin degradation, and cholinesterases (AChE and BChE) and tyrosinase inhibitory activities exhibited by the five seed oils.

	**Green coffee seed oil**	**Coffee seed oil**	**Watermelon seed oil**	**Pumpkin seed oil**	**Broccoli seed oil**
ABTS•(μmolTE/g sample)	7.28^a^ ± 0.08	15.52^c^ ± 0.14	5.92^a^ ± 0.22	5.4^a^ ± 0.45	7.15^a^ ± 0.16
DPPH (IC_50_, μg)	9.75 ± 1.36	7.58 ± 1.21	10.65 ± 1.8	15.27 ± 2.15	10.75 ± 1.87
BSA protective action (IC_50_, μg)^*^	7.79^a^ ± 1.35	8.73^a^ ± 2.11	22.54^c^ ± 2.58	6.25^a^ ± 0.07	6.43^a^ ± 0.12
AChE^*^ inhibition (IC_50_, μg)	14.33^a^ ± 2.26	12.44^a^ ± 2.29	20.72^a^ ± 1.81	17.72^a^ ± 1.72	14.3^a^ ± 1.89
BChE^*^ inhibition (IC_50_, μg)	92.3^d^ ± 5.67	15.7^a^ ± 2.10	139.1^d^ ± 2.58	54.9^c^ ± 1.04	20.7^a^ ± 0.88
Tyrosinase activity inhibition DOPA (IC_50_, μg)^*^	2.77^a^ ± 0.90	10.74^b^ ± 1.45	9^b^ ± 2.05	2^a^ ± 0.61	16.58^c^ ± 1.23

The data of the DPPH test, BSA protective action, AChE, BChE, and tyrosinase activity were expressed as IC_50_, μg. The results of the ABTS test were expressed as μmol TE/g sample. The experiments were performed in triplicate and herein is reported the average (± SD). ^*a^ *p* < 0.5; ^b^*p* < 0.01; ^c^p < 0.001; ^d^*p* < 0.0001 compared with the positive control. ^•^^a^*p* < 0.5; ^b^*p* < 0.01; ^c^*p* < 0.001; ^d^*p* < 0.0001 compared with the average values (ANOVA followed by Dunnett's multiple comparison test).

The DPPH test revealed that all the oils exhibited a remarkable antioxidant capacity. Most oils exhibited excellent IC_50_ values, at most 10.75 μg, except pumpkin seed oil (whose IC_50_ value was 15.27 μg). It is to underline coffee seed oil's most effective antioxidant activity compared with green coffee seed oil, as shown by the DPPH test. The ABTS test was confirmed by the whole results of the DPPH assay. The coffee seed oil showed the best antioxidative performance (15.52 μmol TE/g sample). Green coffee seed oil exhibited even half of the antioxidant activity from the ABTS test. However, the antioxidant activity exhibited by the green coffee seed oil resulted in higher respect than that found by Dong et al. (34), which evidenced an antioxidant activity not higher than 1 μmol TE/gr of product.

On the contrary, the antioxidant activity of the coffee seed oil in our study was inferior to the value (0.267 mmol TE/g sample), observed by Böger et al. (47) during the formulation of skin cream with free or encapsulated roasted coffee oil. No data are present in the scientific literature about the antioxidant activity of broccoli seed oil. Undoubtedly, broccoli seed oil exhibited excellent antioxidant activity, for example, compared with broccoli extracts and seeds (48, 49). The IC_50_ of the watermelon seed oil was 10.65 μg/g, a value different respect to those found by Reziga et al. (50), whose analysis ascertained an antioxidant activity among different Cucurbitaceae seed oils ranging between 64.71 (*Cucumis melo* var. *ananas*) and 159.64 mg/g (*Citrullus lanatus* cv “Crimson”), and corroborated the results obtained by Bagher Hashemi et al. (51). Although pumpkin seed oil was less effective as antioxidant agent (IC_50_ = 15.27 μg), watermelon and pumpkin could be novel sources of edible oils, with promising potential for food applications. In addition, after a prolonged exposition to microwave heating, Bagher Hashemi et al. (51) evidenced that the Iranian watermelon and pumpkin seed oil retained their scavenging capacity of 33.6% and 48.2% of the DPPH-free radicals, respectively.

### *In vitro* inhibitory effect of the oils against the bovine serum albumin degradation

Protein denaturation causes the loss of protein tertiary and secondary structures when external stress acts, such as heat or some compounds, such as strong acids or bases, concentrated inorganic salts, and organic solvents are present in the same environment. The denaturation often gives rise to the loss of biological function, and it can be a cause of inflammation (52). Thus, the measurement of protein denaturation could be a helpful screening assay to detect molecules' anti-inflammatory activity without using animals (53, 54). We determined the action of the oils against the protein denaturation to evaluate their potential *in vitro* anti-inflammatory activity, measuring the amount (μg) of the oils necessary for inhibiting the protein denaturation at 50% (IC_50_). The results are shown in Table 2. All the oils protected the albumin against heat-induced denaturation, with IC_50_ values ranging between 6.25 and 22.54 μg, and the pumpkin seed oil was the most effective (IC_50_ = 6.25 μg). The oils demonstrated a solid *in vitro* anti-inflammatory activity, although weaker than diclofenac (IC_50_= 5.47 μg), used as the control, and some *Prunus* seed oils analyzed by Fratianni et al. (24), which IC_50_ values were not superior to 4.34 μg. Our study confirmed the potential *in vitro* anti-inflammatory activity exhibited by the pumpkin seed oil, although weaker than that observed by Ziaul et al. (55), which ascertained values of IC_50_ not superior to 4 μg for the seed oil from indigenous and hybrid pumpkins. The values of IC_50_ exhibited by the broccoli seed oils confirmed the health benefits of the seeds of Brassicaceae that, for instance, when transformed in flour, showed their potential capacity to modulate gut microbiota also through anti-inflammatory and antioxidant capacities (12). Although exhibiting the weakest anti-inflammatory and antioxidant activities among the five oils tested, our experiments confirmed that the seed of *Citrullus lanatus* had the potential for health effects, further than cosmetics purposes, as hydroalcoholic extract (56) and oil (57). The anti-inflammatory activity shown by the green coffee and coffee seed oils resulted similar (IC_50_=7.79 and 8.73 μg, respectively). Probably, the roasting process did not alter the benefits of the resulting oil, which could be used, like green coffee seed oil, mainly in cosmetics (58).

### *In vitro* cholinesterases and tyrosinase inhibitory activities

Neurodegenerative diseases, leading to a progressive loss in selected areas of the nervous system, are becoming increasingly prevalent worldwide concurrently with the increase in the aging population. In the developmental analysis of such diseases, as in the case of Alzheimer's (AD), the concentrations of the two molecules, acetylcholine and choline, rapidly decline; concurrently, the acetylcholinesterase excessively increases, managing the cellular nerve dysfunction (59) with a subsequent loss of the brain memory and cognitive functions (60). The inhibition of cholinesterase could prevent and treat AD. Most of the studies focused on γ-aminobutyric acid (GABA), the primary inhibitory neurotransmitter of the central nervous system, including the effect of some natural compounds (61). The scarcity of effective treatments to counteract neurodegenerative diseases stimulated interest in the potential neuroprotective activities of plant-derived compounds found abundantly in food and agro-food by-products (62). The beneficial health effects of consuming a traditional Mediterranean diet were described in the Seven Countries Study (63), and then remarked in more investigations (64). People whose diet is rich in fruit and vegetables reduce rates of chronic diseases (i.e., cardiovascular disease, atherosclerosis, some types of cancers, and Alzheimer's disease) and have a higher life expectancy than other worldwide ones (65). Recently, Fratianni et al. investigated the potential *in vitro* effects of some *Citrus* types of honey on ACHe, BCHe, and tyrosinase (66). Vegetable oils, constituting one of the pillars of the Mediterranean diet, can help contrast degenerative diseases (67). They are a rich source of nutraceuticals, which play critical roles in human health, such as linoleic and linolenic acids, monounsaturated fatty acids, phenolic compounds, vitamins, carotenoids, tocopherols, and tocotrienols, which could represent as an alternative for neuroprotection through the regulation of oxidative stress. Olive oil, corn oil, and perilla oil have shown an excellent capacity to alleviate learning and memory dysfunction (68). In this study, we evaluated the *in vitro* potential protective effect of broccoli, coffee, green coffee, pumpkin, and watermelon seed oils against acetylcholinesterase, butyrylcholinesterase, and tyrosinase, involved in neurodegenerative diseases, such as Alzheimer's and Parkinson's diseases. The results are shown in Table 2. Overall, the oils proved to be effective in inhibiting the action of acetylcholinesterase to the extent that the IC_50_, i.e., the quantity necessary to 50% inhibit the enzyme, was never higher than 20.72 μg (watermelon). Coffee seed oil was the most effective (IC_50_= 12.44 μg), followed by green coffee and broccoli seed oils (IC_50_= 14.3 μg) and pumpkin seed oil, whose IC_50_ value turned out to be equal to 17.72 μg.

Opposite to the acetylcholinesterase inhibitory activity, which turned out to be different but still included in a reasonably narrow range, the action of the oils against butyrylcholinesterase resulted in a broader range in terms of IC_50_. Thus, coffee and broccoli seed oils confirmed their better efficacy than other oils in inhibiting butyrylcholinesterase (IC_50_=15.7 μg and 20.7 μg, respectively). On the contrary, watermelon seed oil proved to be less effective (IC_50_= 139.1 μg). Green coffee seed oil, whose action against acetylcholinesterase was similar to that exhibited by the coffee seed oil, was much less effective against BChE so that we needed to use approximately six times the amount of green coffee seed oil to have the same inhibitory strength as coffee seed oil (IC_50_=92.3 μg and 15.7 μg, respectively). Pumpkin seed oil (IC_50_=54.9 μg) exhibited a more effective inhibitory force than watermelon seed oil (IC_50_= 139.1 μg) to inhibit butyrylcholinesterase. Collado-González et al. (69) found that different extra virgin olive oil did not exhibit any *in vitro* inhibitory activity against acetylcholinesterase, although they showed strong α-glucosidase and α-amylase inhibitory activities. Barbosa Filho et al. (70) described olive oil as a natural inhibitor of AChE *in vivo* experiments in rabbits. The oils analyzed in our experiments demonstrated higher cholinesterase inhibitory activity, for example, than that exhibited *in vitro* by some Spanish extra virgin olive oils, which IC_50_, in the case of BuChE and AChE, were never inferior to 298 mg and 483 mg, respectively (71). Pumpkin seed oil confirmed the satisfying results obtained in other studies, such as that of Ahmed et al. (72), which evidenced the neuroprotective effects of the oil against the neurotoxicity due to the presence of the pesticide rotenone in adult male rats. The activity found in pumpkin seed oil can suggest its potential applications in manufacturing some cheese types, which could increase their health and nutritional value, contributing to the prevention of pathologies such as type 2 diabetes, neurodegenerative diseases, and hypercholesterolemia (73). Concerning coffee and green coffee seed oil, previous studies reported a significant effect of green coffee bean extracts in reducing the amyloid-β-protein accumulation in mice used for Alzheimer's disease (73, 74). Consistently, the daily intake of 300–330 mg coffee green extract obtained from green coffee beans for 4–6 months improved cognitive function in healthy humans, which was associated with reduced Aβ42/Aβ40 levels and an increased TTR and apolipoprotein A1 (ApoA1), also involved in Aβ transport (75, 76). Other *in vitro* studies reported that green coffee bean extract, through the presence of chlorogenic acid, inhibits acetylcholinesterase and butyrylcholinesterase, which would increase acetylcholine in the brain (77–79).

The potent inhibitory action of the five oils against tyrosinase should be underlined. The IC_50_ value, i.e., the quantity of oil necessary to inhibit the action of the enzyme by 50%, was even equal to 2.0 μg/ml and 2.77 μg/ml in the case of pumpkin seed oil and green coffee oil, respectively, and it did not go over 16.58 μg/ml (broccoli seed oil). In this case, the behavior exhibited by the two coffee oils was different, with inhibition by the coffee oil approximately five times lower than that exhibited by the green coffee oil. However, it was still very effective since the IC_50_ was evaluated in μg. Our data revealed the excellent *in vitro* efficacy of pumpkin seed oil, even more, effective than observed by Prommaban et al. (80). The seed oil of the other cucurbitaceous analyzed, watermelon, inhibited tyrosinase activity like coffee seed oil (IC_50_ = 9 μg/ml) and was less effective than that observed for the pumpkin oil. Thus, the potential importance that Cucurbitaceae oils can exert is wider than their use in the cosmetic field (81); in fact, since tyrosinase also plays a role in neurodegenerative diseases, after proper *in vivo* studies, they could represent an effective functional tool, for example, when added to the diet of older people (82, 83). The inhibitory efficiency of broccoli seed oil, the weakest among the oils analyzed, confirmed that *Brassica* species could also be a source of important functional properties. Stefanucci et al., for example, reported that the inhibition of tyrosinase activity by pulp and peels of the roots of *Brassica napus* could reach IC_50_ values of 0.79 mg/ml (84). The correlation analysis between the activities evaluated indicates a good correlation between the antioxidant activities evaluated by the DPPH and the ABTS assays (ρ = 0.74) and between the AChE and the BChE inhibitory activities (ρ = 0.78). The AChE inhibition correlated with the anti-inflammatory activity (ρ =0.74); this correlation was similar to that between anti-inflammatory and BChE inhibitory activities (ρ = 0.79). The correlation between the inhibitory activities by the oils and their fatty acids showed that linoleic acid had a braking effect on the AChE inhibition (ρ =0.94), and to a lesser extent, on the BChE inhibitory activity (ρ = 0.58). On the contrary, linolenic acid seemed to limit the inhibitory efficacy exerted by the oils against tyrosinase (ρ =0.78). Instead, stearic acid seemed to influence the antioxidant activity in the DPPH (ρ = −0.72) and ABTS tests (ρ = 0.73).

In this case, the oleic acid, usually an intense antioxidant, proved to inhibit it (ρ = 0.91 with respect to DPPH); with the ABTS test, it turned out (ρ = 0.60). No fatty acids affected positively or negatively the *in vitro* anti-inflammatory activity; linoleic acid seemed to slow down such protective effect (ρ = 0.58).

### Antibacterial activity

Based on the results of the resazurin test (Table 3), two concentrations of seed oils were used to evaluate their potential capacity to inhibit the adhesion process of pathogens that leads to the biofilm and to act on the mature biofilm of these bacteria, a condition conducting to important modifications of pathogens, which, in such a way, become much more resistant to the conventional antibiotics.

**Table 3:** Minimal inhibitory concentration (MIC, μg/ml) of the seed oils, needed to block the metabolic activity of the five bacterial strains.

**Seed oil**	***A. baumannii***	***E. coli***	***L. monocytogenes***	***P. aeruginosa***	***S. aureus***
Broccoli	32 (± 3)^a^	36 (± 2)^c^	32 (± 2)^a^	32 (± 2)^a^	36 (± 2)^a^
Coffee	30 (± 3)^a^	30 (± 2)^b^	35 (± 2)^a^	36 (± 2)^a^	35 (± 2)^a^
Green coffee	36 (± 3)^b^	40 (± 5)^d^	30 (± 5)^a^	36 (± 2)^a^	45 (± 5)^d^
Pumpkin	40 (± 2)^d^	45 (± 2)^d^	30 (± 2)^a^	45 (± 3)^c^	40 (± 2)^b^
Watermelon	45 (± 2)^d^	35 (± 4)^c^	48 (± 2)^d^	40 (± 3)^b^	45 (± 3)^d^
Tetracycline	30 (± 2)	25 (± 3)	33 (± 2)	34 (± 2)	36 (± 2)

Tetracycline was used as a standard drug. Results are expressed as the mean of three experiments ± SD. ^a^*p* < 0.5; ^b^*p* < 0.01; ^c^*p* < 0.001; ^d^*p* < 0.0001 (ANOVA followed by Dunnett's multiple comparison test).

We used these bacteria considering also their role in some pathologies affecting the central nervous system.

The results of the inhibitory activity of the seed oils on immature and mature biofilm are shown in Table 4.

**Table 4:** Inhibitory activity of the seed oils on the bacterial biofilm formation, at 10 and 20 μg/ml against *A. baumannii, E. coli, L. monocytogenes, P. aeruginosa*, and *S. aureus*.

	***A. baumannii***	***E. coli***	***L. monocytogenes***	***P. aeruginosa***	***S. aureus***
**CV - Time 0**					
Broccoli 10 μg/ml	0^a^ (± 0.00)	0^a^ (± 0.00)	0^a^ (± 0.00)	0^a^ (± 0.00)	2.47^a^ (± 0.12)
Broccoli μg/ml 20	6.72^a^ (± 0.87)	3.78^a^ (± 0.12)	5.95^a^ (± 0.35)	0^a^ (± 0.00)	4.66^a^ (± 0.14)
Coffee 10 μg/ml	8.22^a^ (± 0.74)	3.44^a^ (± 0.13)	1.98^a^ (± 0.07)	0^a^ (± 0.00)	36.24^c^ (± 0.24)
Coffee 20 μg/ml	14.02^b^ (± 0.15)	7.38^a^ (± 0.54)	46.46^c^ (± 2.24)	0^a^ (± 0.00)	39.91^c^ (± 2.57)
Green coffee 10 μg/ml	7.66^a^ (± 0.57)	8.49^a^ (± 0.57)	3.99^a^ (± 0.11)	0^a^ (± 0.00)	67.54^d^ (± 4.56)
Green coffee20 μg/ml	13.12^b^(± 0.57)	12.71^b^(± 1.03)	40.99^c^ (± 2.21)	0^a^ (± 0.00)	76.10^d^ (± 4.31)
Pumpkin 10 μg/ml	11.11^b^ (± 0.57)	12.87^b^ (± 0.57)	31.85^c^ (± 1.67)	22.86^b^ (± 1.57)	51.59^d^ (± 2.13)
Pumpkin 20 μg/ml	12.21^b^ (± 0.57)	15.91^b^ (± 0.67)	50.07^d^ (± 1.13)	34.41^c^ (± 1.67)	55.45^d^ (± 2.57)
Watermelon 10 μg/ml	12.53^b^ (± 0.57)	7.85^a^ (± 0.57)	0^a^ (± 0.00)	0^a^ (± 0.00)	6.41^a^ (± 0.67)
Watermelon 20 μg/ml	16.42^b^ (± 1.02)	29.00^c^ (± 1.57)	47.70^c^ (± 2.44)	21.38^b^ (± 1.57)	11.35^b^ (± 0.57)
**CV - Time 24 h**					
Broccoli 10 μg/ml	0.29^a^ (± 0.09)	0^a^ (± 0.00)	0^a^ (± 0.00)	0^a^ (± 0.00)	15.52^b^ (± 0.57)
Broccoli μg/ml 20	9.03^a^ (± 0.30)	9.53^a^ (± 0.00)	53.41^d^ (± 3.57)	0^a^ (± 0.00)	40.50^c^ (± 1.67)
Coffee 10 μg/ml	18.76^b^ (± 1.67)	3.49^a^ (± 0.57)	0^a^(± 0.00)	0^a^ (± 0.00)	56.70^d^ (± 4.13)
Coffee 20 μg/ml	42.66^c^ (± 2.67)	15.44^b^ (± 1.13)	0^a^ (± 0.00)	0^a^ (± 0.00)	59.22^d^ (± 2.23)
Green coffee 10 μg/ml	12.42^b^ (± 1.25)	8.28^a^ (± 0.07)	55.99^d^ (± 4.23)	0^a^ (± 0.00)	0^a^ (± 0.00)
Green coffee 20 μg/ml	53.72^d^ (± 4.57)	12.10^b^ (± 0.89)	56.95^d^ (± 4.55)	0^a^(± 0.00)	13.41^b^ (± 1.03)
Pumpkin 10 μg/ml	0^a^ (± 0.00)	0^a^ (± 0.00)	21.43^b^ (± 1.67)	0^a^ (± 0.00)	52.39^d^ (± 4.23)
Pumpkin 20 μg/ml	17.59^b^ (± 1.04)	6.82^a^ (± 0.57)	26.95^c^ (± 1.45)	0^a^ (± 0.00)	57.39^d^ (± 4.57)
Watermelon 10 μg/ml	11.40^b^ (± 1.04)	0^a^ (± 0.00)	28.33^c^ (± 1.57)	0^a^ (± 0.00)	28.21^c^ (± 1.67)
Watermelon 20 μg/ml	15.26^b^ (± 1.44)	0.77^a^ (± 0.02)	33.75^c^ (± 2.25)	0^a^ (± 0.00)	66.92^d^ (± 5.44)

The results are expressed as the mean of three experiments ± SD. ^a^*p* < 0.5; ^b^*p* < 0.01; ^c^*p* < 0.001; and ^d^*p* < 0.0001 compared with the control (ANOVA followed by Dunnett's multiple comparison test).

The coffee seed oil (CSO) efficacy in inhibiting the sessile cells of *A. baumannii* and *L. monocytogenes* was practically similar to the tested concentrations. Green coffee seed oil (GCSO) exhibited a more potent inhibition compared with the coffee seed oil (CSO) when it was tested against *S. aureus*, both at 10 μg/ml (67.54% and 36.24%, respectively for GCSO and CSO) and 20 μg/ml (76.10 and 39.91%, respectively). The two oils showed mild inhibitory activity vs. *E. coli*, which did not exceed 12.71%. Instead, these oils were completely ineffective *vs. Ps. aeruginosa*. The activity of these seed oils increased when in contact with the mature biofilm of *A. baumannii*. Their inhibitory efficacy went from 13.12% to 53.72% (GCSO) and from 14.02% to 42.66% (CSO), increasing more than three times compared with the efficacy in counteracting the adhesion of the cells of this strain. This aspect is relevant considering the difficulties of eradicating a mature biofilm when a microorganism has transformed its physiology and increased its virulence and antibiotic resistance. While the effectiveness of the GCSO against *S. aureus* decreased, dropping to 13.41%, the inhibition of the CSO against the mature biofilm of this microorganism increased, reaching 59.22%. The inhibitory efficacy of the GCSO against the mature biofilm of *L. monocytogenes* increased up to 56.95%; on the contrary, CSO resulted ineffectively. The behavior of the GCSO remained virtually unchanged *vs. E. coli* (inhibition on mature biofilm of 12.10%), while CSO doubled its inhibitory activity (from 7.98 % to 15.44 %). The seed oils of the two Cucurbitaceae, watermelon seed oil (WMSO) and pumpkin seed oil (PSO), acted mainly in inhibiting the adhesion process of *L. monocytogenes* (47.70 % and 50.07 %, respectively). Their inhibition *vs. S. aureus* was not similar: WMSO inhibition did not exceed 11.35%, while PSO showed 51.59% and 55.45% inhibition at 10 μg/ml and 20 μg/ml, respectively. *P. aeruginosa*, contrary to what we verified with the two coffee seed oils, was sensitive to WMSO and PSO, with inhibition of the adhesion process by 21.38% and 34.41%, respectively. These seed oils were also effective against *E. coli*, with inhibition of 29.0% (WMSO) and 15.91% (PSO). The inhibitory effect of these oils decreased on the mature biofilm of *E. coli* and *L. monocytogenes*. At the same time, their action remained unchanged or even increased in countering the mature biofilm of *S. aureus*, with inhibition of 57.39 % and 66.92%, respectively, for PSO and WMSO. These oils, however, were utterly ineffective in counteracting the mature biofilm of *P. aeruginosa*. The broccoli seed oil (BSO) showed considerable weakness in counteracting the adhesion process of the pathogenic strains and inhibiting their mature biofilm, except for the activity against the mature biofilm of *S. aureus* (where the inhibition passed from 4.66% to 40.50%) and *L. monocytogenes* (when the inhibitory effectiveness increased from 5.95% to 53.41%). It opens the possibility of using this oil in the case of particularly resistant infections, such as that of *S. aureus* and *L. monocytogenes*. Therefore, its utilization in the food and health sectors could be considered.

The test performed to evaluate the effect of the seed oils on bacterial biofilm metabolism (Table 5) gave some underlining results. In the test carried out on the immature biofilm, the seed oils were very active against the metabolism of *S. aureus* sessile cells, with inhibition percentages that reached 80.54% (PSO), confirming literature data on the biofilm produced by *S. aureus* (85) or *P. aeruginosa* (86). A minor activity was evident when the oils were in contact with *E. coli*, whose highest inhibitory activity was approximately 48%. Except for CSO, which was completely ineffective, all the oils inhibited the metabolism of the sessile cells of *L. monocytogenes*, as evidenced by other seed oils (24). GCSO was particularly effective: it inhibited cell metabolism by 75.65% used 10 μg/ml; therefore, its inhibitory action was 80.45% at 20 μg/ml; PSO, WMSO, and BSO also showed inhibition of 68.02%, 54.50%, and 58.82%, respectively. It demonstrates again that other mechanisms could be activated, not linked to the metabolic pathway, which we intend to study in future. In the MTT tests carried out on mature biofilm, all the oils were active on the metabolism of *A. baumannii* cells, albeit in a less effective way than that observed in the adhesion process, with inhibition percentages ranging between 17.76% (GCSO) and 39.50% (CSO). The oils were ineffective in inhibiting the metabolism of *P. aeruginosa* and *L. monocytogenes*. The two Cucurbitaceae oils exhibited an inhibitory action of up to 27.29%. Except for CSO and PSO, the oils also were more effective in inhibiting the metabolism of *S. aureus* sessile cells in mature biofilms. In this case, the most effective seed oil was GCSO, which, already at 10 micrograms/ml, determined an inhibition of 36.87% (41.20% at 20 μg/ml). The inhibitory effect could be derived from a set of events that do not concern only the metabolic pathway of bacterial cells.

**Table 5:** Inhibitory activity of the seed oils on the bacterial cell metabolism within the biofilm, at 10 μg/ml and 20 μg/ml against *A. baumannii, E. coli, L. monocytogenes, P. aeruginosa*, and *S. aureus*.

	***A. baumannii***	***E. coli***	***L. monocytogenes***	***P. aeruginosa***	***S. aureus***
**MTT - Time 0**					
Broccoli 10 μg/ml	47.95^d^ (± 0.28)	29.03^c^ (± 0.22)	49.38^d^ (± 0.42)	0^a^ (± 0.00)	76.30^d^ (± 0.67)
Broccoli 20 μg/ml	70.92^d^ (± 0.68)	10.01^a^ (± 0.39)	58.82^d^ (± 0.52)	0^a^ (± 0.00)	72.48^d^ (± 0.72)
Coffee 10 μg/ml	0^a^ (± 0.00)	0^a^ (± 0.00)	0^a^ (± 0.00)	0^a^ (± 0.00)	66.26^d^ (± 4.24)
Coffee 20 μg/ml	27.05^c^ (± 0.33)	10.76^a^ (± 0.08)	0^a^ (± 0.00)	0^a^ (± 0.00)	70.73^d^ (± 3.57)
Green coffee 10 μg/ml	0^a^ (± 0.00)	9.08^a^ (± 0.33)	75.65^d^ (± 5.67)	0^a^ (± 0.00)	48.90^d^ (± 3.24)
Green coffee 20 μg/ml	54.11^d^ (± 3.44)	24.52^c^ (± 1.57)	80.45^d^ (± 7.05)	0^a^ (± 0.00)	65.42^d^ (± 5.34)
Pumpkin 10 μg/ml	0^a^ (± 0.00)	8.39^a^ (± 1.72)	50.96^d^ (± 3.32)	14.87^b^ (± 1.13)	62.68^d^ (± 5.67)
Pumpkin 20 μg/ml	12.46^b^ (± 1.02)	47.58^d^ (± 3.97)	68.02^d^ (± 5.44)	66.34^d^ (± 5.67)	80.54^d^ (± 4.22)
Watermelon 10 μg/ml	0^a^ (± 0.00)	11.79^a^(± 1.02)	20.29^b^ (± 2.22)	0^a^ (± 0.00)	61.31^d^ (± 7.08)
Watermelon 20 μg/ml	54.70^d^ (± 3.98)	48.01^d^ (± 4.04)	54.50^d^ (± 4.67)	0^a^ (± 0.00)	66.58^d^ (± 5.21)
**MTT - Time 24 h**					
Broccoli 10 μg/ml	14.16^b^ (± 1.54)	0^a^ (± 0.00)	0^a^ (± 0.00)	0^a^ (± 0.00)	0^a^ (± 0.00)
Broccoli 20 μg/ml	26.96^c^ (± 2.02)	0^a^ (± 0.00)	0^a^ (± 0.00)	0^a^ (± 0.00)	24.89^c^(± 3.32)
Coffee 10 μg/ml	0^a^ (± 0.00)	0^a^ (± 0.00)	0^a^ (± 0.00)	0^a^ (± 0.00)	0^a^ (± 0.00)
Coffee 20 μg/ml	39.50^d^ (± 4.05)	0^a^ (± 0.00)	0^a^ (± 0.00)	0^a^ (± 0.00)	0^a^ (± 0.00)
Green coffee 10 μg/ml	6.05^a^ (± 1.05)	0^a^ (± 0.00)	0^a^ (± 0.00)	0^a^ (± 0.00)	36.87^d^ (± 2.57)
Green coffee 20 μg/ml	17.76^b^ (± 1.57)	7.50^b^ (± 0.75)	0^a^ (± 0.00)	0^a^ (± 0.00)	41.40^d^ (± 3.98)
Pumpkin 10 μg/ml	0^a^ (± 0.00)	0^a^ (± 0.00)	0^a^ (± 0.00)	0^a^ (± 0.00)	0^a^ (± 0.00)
Pumpkin 20 μg/ml	21.15^c^ (± 1.57)	0^a^ (± 0.00)	27.29^c^ (± 0.00)	1.84^a^ (± 0.00)	0^a^ (± 0.00)
Watermelon 10 μg/ml	19.88^c^ (± 1.06)	0^a^ (± 0.00)	0^a^ (± 0.00)	0^a^ (± 0.00)	0^a^ (± 0.00)
Watermelon 20 μg/ml	34.92^d^ (± 2.67)	0^a^ (± 0.00)	7.23^b^ (± 0.65)	0^a^ (± 0.00)	20.00^c^ (± 1.55)

The results are expressed as the mean of three experiments ± SD. ^a^*p* < 0.5; ^b^*p* < 0.01; ^*c*^*p* < 0.001; and ^d^*p* < 0.0001 compared with the control (ANOVA followed by Dunnett's multiple comparison test).

In some cases, the significant inhibitory efficacy exerted by the oils on the immature biofilm corresponded to equally effective action on its metabolism. For example, in the case of *E. coli*, at 20 μg/ml, the correlation coefficient ρ = of 0.87 was registered between the crystal violet (CV) and the MTT tests. In the other cases, however, the correlation was not very evident: in the CV and MTT tests conducted against *A. baumannii* at 10 μg/ml, the influence was even the opposite (ρ = −0.91); we also observed similar behavior in the case of *E. coli* (ρ = −0.73). We observed often a similar behavior when the test was performed on the mature bacterial biofilms: while with the lowest concentration of oils, their effect on the metabolism of the biofilm of *A. baumannii* was slightly braking (ρ = −0.13); as the quantity of oils used increased, as the braking effect increased (ρ = −0.51). Considering the composition of fatty acids and the impact of the oils on the biofilm, we might underline some points: palmitic acid seemed to exert an influence on the inhibitory capacity against the immature biofilm *vs. S. aureus* both at 10 μg/ml and 20 μg/ml (ρ = 0.76 and ρ = 0.78, respectively); the effect of palmitic acid seemed to affect also the inhibitory capacity exhibited by the oils on the mature biofilm of *A. baumannii* (ρ = 0.99 and ρ = 0.98, at 10 μg/ml and 20 μg/ml, respectively). The effects of stearic acid on the mature biofilm of *A. baumannii* (ρ = 0.95 and ρ = 0.88, with 10 20 μg/ml and 20 μg/ml, respectively) should also be noted. On the contrary, oleic acid seemed to slow the inhibition of the oils against this microorganism: thus, the correlation coefficient ranged from ρ =0.33 to ρ = 0.22 at 10 μg/ml and 20 μg/ml in the test performed on immature biofilm, and it resulted negative (ρ = −0.69 and ρ = −0.60) in the test performed on the mature biofilm, conversely to that observed by Khadke et al. (87). Oleic acid seemed to influence the inhibitory capacity exerted by the oils against *L. monocytogenes* at the lower concentration (ρ = 0.88). However, as the quantity of oils used in the CV test increased, its influence decreased drastically (ρ = 0.45). On the contrary, its effect of inhibiting the immature biofilm produced by *E. coli* seemed to grow with the increasing quantity of oils so that it passed from ρ = 0.49 at 10 μg/ml to ρ= 0.82 at 20 μg/ml. In the case of *S. aureus*, the effect was weaker (ρ = 0.55 and 0.50, at 10 μg/ml and 20 μg/ml, respectively). These data disagree with Stenz et al. (88), which found a vigorous inhibitory activity of oleic acid against the biofilm formation of *S. aureus*. The influence of single fatty acids on biofilm metabolism was different. In addition, in this case, considering only the circumstances in which a specific fatty acid was present in the majority of the oils and the percentage of inhibition was at least four times in five, oleic acid appeared to influence *P. aeruginosa* biofilm metabolism in the case of the immature biofilm (ρ =0.93 with both 10 μg/ml and 20 μg/ml). The influence of oleic acid seemed to increase with the increase of the dose of oils tested in the MTT test against *S. aureus*, passing from a low correlation coefficient (ρ = 0.25) at 10 μg/ml to a high correlation coefficient (ρ = 0.89) at 20 μg/ml.

## Conclusion

The results exhibited by the oils in the antioxidant tests, their fatty acid composition, and indexes of atherogenicity and thrombogenicity, as well as their potential *in vitro* capacity of inhibiting the activity of the three enzymes involved in some of the neurodegenerative diseases, will be helpful in better defining their future *in vivo* study to evaluate their potential activity on human health. Their inhibitory action against the bacterial biofilm and metabolism pathogens, also involved in neurodegenerative diseases and whose biofilm becomes increasingly problematic to remove, could address new applications in the health fields. Furthermore, the *in vitro* oil's anti-inflammatory activity also could give them potential relevance as natural anti-inflammatory agents through their regular supply in a dietary plan after appropriate *in vivo* studies. Finally, the *in vitro* properties exhibited by these oils could be taken into consideration also in food science and technology. Some of these oils, for instance, could be also used as ingredients to prolong the shelf life of foods (particularly susceptible to oxidative events and, in the case of fresh foods, to fight the occurrence of food contaminants, such as *E.coli* or *L. monocytogenes)* or to create new and innovative functional foods, after appropriate study also including the quality, the sensorial, and the safe aspects.

## Data availability statement

The raw data supporting the conclusions of this article will be made available by the authors, without undue reservation.

## Author contributions

FN and FF: conceptualization and methodology. Ad'A, GA, and VDF: software. FF, FN, VDF, and GA: investigation. FN, RC, VDF, and FF: writing—original draft preparation. FN: supervision and funding acquisition. All the authors have read and agreed to the published version of the manuscript.
